# Bilateral superficial peroneal nerve compression secondary to peroneus muscle herniations mimicking spinal claudication

**DOI:** 10.1016/j.radcr.2026.06.052

**Published:** 2026-07-13

**Authors:** Sarah Eustace, Marion Hanley, David Ryan, John Hynes, Eoin Kavanagh

**Affiliations:** Department of Radiology, Cappagh National Orthopaedic Hospital Finglas Dublin 11, Dublin, Ireland

**Keywords:** Superficial peroneal nerve, Muscle herniation, Peroneus longus, Spinal claudication, Peripheral nerve entrapment, MRI

## Abstract

Muscle herniation is an uncommon musculoskeletal condition that may present with non-specific lower limb symptoms and, in rare cases, result in peripheral nerve compression. We report the case of a 32-year-old woman who presented with a two-year history of progressive exertional bilateral lateral calf pain and dysesthesia initially suspected to represent neurogenic claudication. Lumbar spine magnetic resonance imaging (MRI) demonstrated no evidence of spinal canal stenosis, intervertebral disc herniation or nerve root compression. Subsequent MRI of both calves performed on a 1.5-T MRI system revealed bilateral focal fascial defects (measuring approximately 5-8 mm) within the lateral compartments with herniation of the peroneus longus muscles and associated compression of the superficial peroneal nerves at their fascial exit points. No vascular compression or alternative cause of nerve entrapment was identified. The patient underwent bilateral fasciotomy with superficial peroneal nerve decompression, resulting in complete resolution of symptoms. This case highlights an uncommon cause of bilateral exertional leg pain mimicking spinal claudication and emphasizes the importance of considering peripheral nerve entrapment secondary to muscle herniation when spinal investigations are inconclusive. MRI plays a key role in identifying fascial defects, muscle herniation and associated nerve compression, thereby facilitating appropriate surgical management.

## Introduction

Muscle herniation is an uncommon musculoskeletal condition characterized by protrusion of muscle tissue through a defect in the surrounding fascia. Although frequently asymptomatic, muscle herniations may present with pain, swelling, exercise-induced discomfort or neurological symptoms when adjacent neurovascular structures become compressed [[1], [2], [3]]. The lower limb is the most common anatomical location for muscle herniation and these lesions are most frequently encountered in adolescents and young adults [1,3,4]. The tibialis anterior muscle is most commonly affected owing to its superficial location and susceptibility to trauma and fascial weakness [1,3].

Peroneal compartment muscle herniations are considerably less common than tibialis anterior herniations and are infrequently reported in the literature [3,5]. The superficial peroneal nerve (SPN) exits the lateral compartment through the crural fascia in the distal third of the leg, rendering it vulnerable to entrapment at sites of fascial abnormality [6,7]. SPN entrapment may result in pain, paresthesia, dysesthesia and sensory disturbance affecting the anterolateral leg and dorsum of the foot. In some patients, symptoms may mimic lumbar radiculopathy or neurogenic claudication, creating a significant diagnostic challenge [6,7].

Dynamic ultrasonography and magnetic resonance imaging (MRI) are recognized as the principal imaging modalities for evaluating muscle herniation. Ultrasonography permits real-time assessment during muscle contraction, whereas MRI provides superior anatomical characterization of fascial defects, muscle herniation, associated nerve compression and alternative causes of symptoms [[8], [9], [10]].

We report a rare case of bilateral SPN compression secondary to peroneus longus muscle herniation presenting with symptoms initially attributed to spinal claudication. This case highlights an important diagnostic pitfall and emphasizes the role of targeted peripheral imaging in patients with persistent symptoms and normal spinal investigations.

## Case presentation

A 32-year-old woman presented with a two-year history of progressively worsening bilateral lateral calf pain associated with dysesthesia. The symptoms were exertional in nature, initially occurring after prolonged walking but gradually progressing to occur after walking distances as short as approximately 90 meters. The patient also reported symptom exacerbation with prolonged standing. Symptoms were consistently relieved with rest.

The patient had no significant past medical or surgical history. There was no family history of neuromuscular, connective tissue or peripheral nerve disorders. She denied lower back pain, lower limb trauma and previous lower limb surgery. There was no history suggestive of vascular claudication.

Clinical examination demonstrated altered sensation over the anterolateral aspects of both lower legs. Motor examination revealed no weakness and there was no evidence of foot drop. Dorsalis pedis and posterior tibial pulses were palpable and symmetrical bilaterally. No palpable masses were identified in either calf at rest.

Routine laboratory investigations were within normal limits and were not contributory to the diagnosis.

Given the exertional nature of the symptoms and their distribution, neurogenic claudication secondary to lumbar spinal pathology was initially suspected. MRI of the lumbar spine demonstrated no evidence of spinal canal stenosis, intervertebral disc herniation or nerve root compression.

Due to the persistence of symptoms and normal lumbar imaging, further evaluation was undertaken. MRI of both calves was performed on a 1.5-T MRI system using axial and coronal T1-weighted and T2-weighted sequences. Imaging demonstrated bilateral focal defects measuring approximately 5-8 mm in the deep fascia of the lateral compartments. Through these defects, the peroneus longus muscles were seen to herniate into the subcutaneous tissues. At the level of the fascial defects, the superficial peroneal nerves were visualized in close proximity to the herniated muscle and appeared compressed (Fig. 1A–D).

**Fig. 1:**
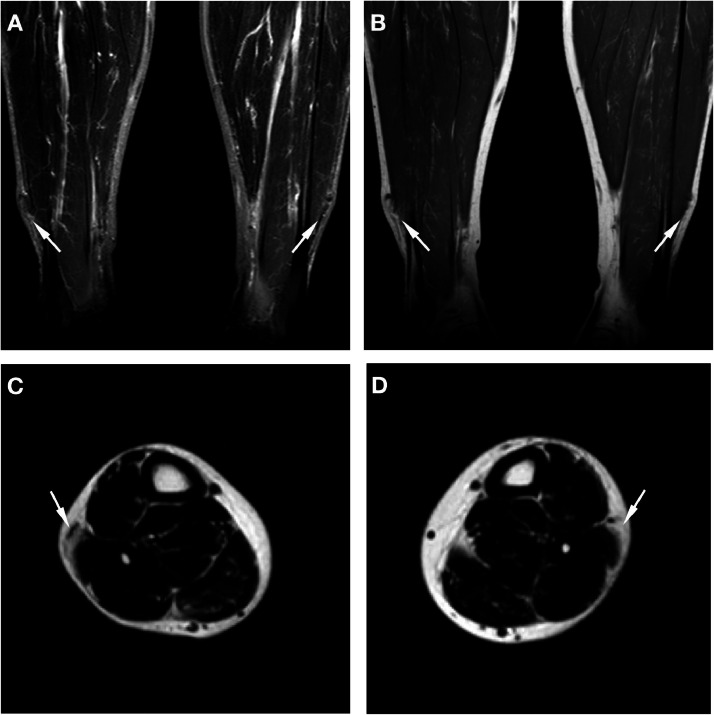
(A) Coronal T2-weighted MRI demonstrating bilateral focal fascial defects within the lateral compartments with protrusion of the peroneus longus muscles (white arrows). Associated superficial peroneal nerve compression is present at the fascial exit points. (B) Coronal T1-weighted MRI confirming bilateral peroneus longus muscle herniation through focal fascial defects (white arrows). (C) Axial T1-weighted MRI of the right calf demonstrating focal peroneus longus muscle herniation with adjacent superficial peroneal nerve compression (white arrow). (D) Axial T1-weighted MRI of the left calf demonstrating focal peroneus longus muscle herniation with adjacent superficial peroneal nerve compression (white arrow).

No alternative cause of superficial peroneal nerve compression was identified. Specifically, there was no evidence of lipoma, peripheral nerve sheath tumor, ganglion cyst, venous malformation, venous engorgement or vascular compression.

These imaging findings were considered consistent with bilateral superficial peroneal nerve compression secondary to peroneus longus muscle herniation and correlated closely with the patient’s symptoms.

Initial management consisted of activity modification, physiotherapy, and analgesia over a period of six months. Due to persistent symptoms, multidisciplinary discussion was undertaken and the patient underwent bilateral targeted fasciotomy with decompression of the superficial peroneal nerves. The procedure was performed without complication.

At postoperative follow-up, the patient reported complete resolution of symptoms, including both pain and dysesthesia. She was able to return to normal activities without limitation. Postoperative imaging was not performed because complete clinical resolution was achieved and there was no clinical suspicion of recurrent nerve compression or postoperative complications.

## Discussion

Muscle herniations occur through focal defects in the deep fascia and may be congenital or acquired. Acquired herniations are typically associated with trauma, repetitive muscular stress, increased intracompartmental pressure or chronic fascial weakening, whereas congenital herniations arise from developmental defects within the fascial envelope [1,2].

Although many muscle herniations remain asymptomatic, symptomatic lesions may cause pain, swelling, cramping, sensory disturbance or neurological dysfunction secondary to compression of adjacent neurovascular structures [[1], [2], [3]]. The present case is unusual because bilateral peroneus longus muscle herniation resulted in compression of both superficial peroneal nerves, producing symptoms that closely mimicked neurogenic claudication.

Entrapment neuropathies of the superficial peroneal nerve are uncommon and frequently under-recognized. Fortier et al. highlighted that SPN entrapment may mimic lumbar radiculopathy owing to overlapping sensory symptoms and that delayed diagnosis is common when clinical attention is focused on spinal pathology [6]. In the present case, normal MRI of the lumbar spine prompted further investigation of the peripheral nervous system, ultimately identifying the true source of symptoms.

The differential diagnosis included lumbar radiculopathy, chronic exertional compartment syndrome, vascular claudication, lipoma, schwannoma, ganglion cyst, venous malformation, venous engorgement and other causes of peripheral nerve compression. MRI excluded these alternative diagnoses by demonstrating bilateral focal fascial defects with protrusion of the peroneus longus muscles and associated SPN compression.

Although dynamic ultrasonography is frequently considered a first-line imaging modality for muscle herniation because it permits real-time assessment during muscle contraction, it was not performed in the present case. No palpable soft tissue mass was identified clinically and the primary diagnostic concern was neurogenic claudication. MRI was therefore prioritized because it enabled simultaneous assessment of fascial integrity, musculature, peripheral nerves and alternative causes of the patient’s symptoms. In this case, MRI clearly demonstrated the fascial defects, peroneus longus muscle herniation and associated superficial peroneal nerve compression.

Foresti et al. described a similar case of superficial peroneal nerve compression secondary to peroneal muscle herniation diagnosed using ultrasonography and MRI [11]. Their report emphasized the importance of considering muscle herniation in patients with otherwise unexplained sensory symptoms affecting the SPN distribution. Our case expands upon these observations by demonstrating bilateral disease presenting as spinal claudication.

Management of muscle herniation depends on symptom severity. Most asymptomatic or mildly symptomatic patients may be managed conservatively with activity modification, physiotherapy, compression therapy and anti-inflammatory medication [12]. Surgical intervention is generally reserved for persistent pain, neurological symptoms, functional impairment or failure of conservative management.

Several operative techniques have been described, including direct fascial repair, mesh reconstruction, periosteal grafting and longitudinal fasciotomy [1,12]. Recent studies continue to support longitudinal fasciotomy as a safe and effective option, particularly when associated nerve compression is present [13,14]. Matsumoto et al. demonstrated favorable outcomes following decompression of peroneal nerve entrapment syndromes, while Nuelle et al. reported successful surgical treatment of symptomatic peroneal muscle herniation with restoration of function and symptom resolution [13,14]. Consistent with these findings, our patient experienced complete resolution of symptoms following bilateral fasciotomy and SPN decompression.

A limitation of this report is that postoperative MRI was not performed because complete clinical resolution was achieved and there was no indication for further imaging.

## Conclusion

Bilateral superficial peroneal nerve compression secondary to peroneus longus muscle herniation is an uncommon but important differential diagnosis in patients presenting with exertional lower limb pain suggestive of spinal claudication. When lumbar spine imaging is normal, clinicians should consider peripheral causes of nerve entrapment. MRI is particularly valuable in identifying fascial defects, muscle herniation and associated nerve compression, thereby guiding definitive surgical management.

## Patient consent

Written informed consent and ethics approval was obtained for this case report.
